# Nobody dares stopping clinical research, not even COVID-19

**DOI:** 10.1038/s41523-021-00249-1

**Published:** 2021-04-08

**Authors:** Andrea Malfettone, Serena Di Cosimo, José Manuel Pérez-García, Alicia García, Miguel Sampayo-Cordero, Leonardo Mina, Carolina Herrero, Antonio Llombart-Cussac, Javier Cortés

**Affiliations:** 1 Medica Scientia Innovation Research (MEDSIR), Ridgewood, NJ USA; 2 Medica Scientia Innovation Research (MEDSIR), Barcelona, Spain; 3 Biomarkers Unit, Department of Applied Research and Technological Development, Fondazione IRCCS Istituto Nazionale dei Tumori, Milano, Italy; 4 International Breast Cancer Center (IBCC), Quiron Group, Barcelona, Spain; 5 Hospital Arnau de Vilanova, Valencia, Spain; 6 Universidad Católica de Valencia San Vicente Mártir, Valencia, Spain; 7 Vall d´Hebron Institute of Oncology (VHIO), Barcelona, Spain

**Keywords:** Medical research, Health care

## Abstract

In the global health emergency caused by the COVID-19, clinical trial management has proven to be critical for the pharmaceutical industry, sponsors, and healthcare professionals. Our experience as a sponsor managing interventional oncology clinical studies has provided us with some data and insights. Though limited by sample size, our data emphasize the importance of quickly adopting measures that first prioritize patient safety and data validity, then consider contingency measures such as telemedicine, virtual medical review, and remote monitoring. Successful adaptations of healthcare and patient management in response to COVID-19 have been fundamental to ensuring continuing clinical cancer research.

Clinical research trials can be lifesaving for many patients. They provide vital information necessary for continuing the search for a cure, new medications, devices, and therapies. Because of the rapidly escalating outbreak of the respiratory coronavirus disease 2019 (COVID-19), the capacities of the healthcare systems in the affected countries have been put under pressure, and they have quickly reached their limits. In addition to the existing structural and clinical barriers to clinical trial participation, the COVID-19 public health emergency can negatively influence how clinical trials are conducted and impose additional obstacles to patient enrollment and ongoing monitoring in investigative research. With this in mind, international and national regulatory agencies have approved contingency measures that both sponsors and principal investigators should consider so that the healthcare of the trial participants is ensured, protecting their safety, welfare, rights, and well-being, and secondarily to preserve the trial as much as possible^1–8^.

MEDSIR is a private company based in Europe and the US that is dedicated to the design and management of strategic clinical trials. Since its founding in 2012, MEDSIR has sponsored or collaborated in 31 trials in oncology: studies on early (*N* = 4) and metastatic (*N* = 19) breast cancer, lung cancer (*N* = 3), prostate cancer (*N* = 2), endometrial carcinoma (*N* = 2), and penile carcinoma (*N* = 1).

On March 11, 2020 when the World Health Organization formally declared the COVID-19 outbreak a pandemic, 10 clinical trials sponsored by MEDSIR were actively recruiting patients. A series of extraordinary measures were implemented to ensure both the safety of office staff, and well-being of trial participants, and the integrity of the trials.

## Measures implemented for staff

Protecting our workforce was our highest priority, followed closely by continuing to foster and support clinical research. On March 14, a nationwide lockdown of the Spanish government ordered all non-essential workers to stay at home in an effort to reduce the risk of contagion and release the overstretched intensive care units. As a consequence, a number of pragmatic and harmonized actions were taken by MEDSIR to allow flexibility and procedural simplifications. One example is flexible work-at-home options for the entire employee staff (*N* = 46).

To mitigate operational challenges and guarantee the safety of our staff, we reinforced pre-existing technology-based interventions aimed at limiting on-site monitoring visits while maintaining compliance with Good Clinical Practice. Specifically, clinical trial monitors (*N* = 29) developed ways of remotely overseeing trial activities and patients’ well-being within the study by employing phone calls, and remote review of medical records for all the clinical trials (*N* = 14) deserving monitoring during the pandemic. As a result, to date all of the clinical trials have been successfully monitored.

## Measures implemented for clinical trial participants

**Fig. 1:**
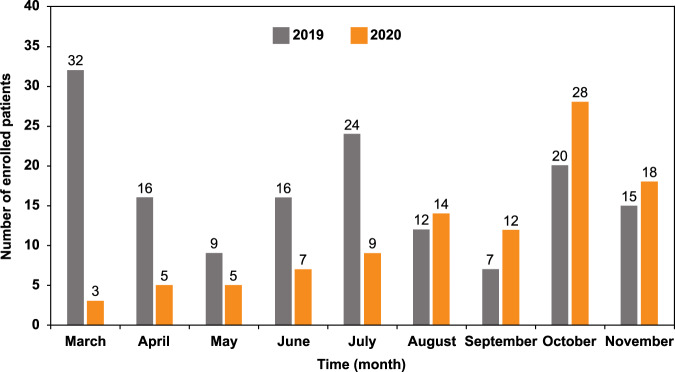
Bar chart showing changes in the monthly accrual of cancer-focused, actively recruiting clinical trials from March through November 2019 compared to the same period in 2020. Each bar represents the total number of enrolled patients by month.

An internal MEDSIR survey was fostered to assess the effect of measures implemented and actions taken for ongoing clinical trials during the first 9 months of the pandemic (Table 1). Information was collected from ad hoc reports that MEDSIR prepared and compiled specifically for each trial in the aftermath of the COVID-19 outbreak. Henceforth, all clinical trials with one exception involved the treatment of patients with solid tumors, primarily breast cancer (*N* = 10); and were based on immunotherapy (*N* = 4), chemotherapy (*N* = 3), targeted therapy (*N* = 6), and hormone therapy (*N* = 1). As cancer patients have been described as highly vulnerable to COVID-19 morbidity and mortality, and have increased risk of all infections, especially after treatment with toxic chemotherapy or surgery, it is not surprising that patient enrollment was halted in all sites (*N* = 1 study based on immunotherapy) or in part of the sites (*N* = 1 study with hormone therapy, *N* = 1 with targeted-therapy, *N* = 1 with immunotherapy, and *N* = 1 with chemotherapy), and the site activation was delayed in additional 2 trials (*N* = 1 with immunotherapy and *N* = 1 with targeted-therapy). Patient enrollment was slowed down during pandemic and the accrual rate of actively recruiting clinical trials in 2020 showed a ~30% decline in comparison to 2019, especially during first half of 2020 when tighter nationwide restrictions were adopted to stop the spread of the SARS-CoV-2 (Fig. 1). The reduction of clinical trial accrual was somewhat expected and in line with National Cancer Institute|National Institutes of Health data^6^.

**Table 1:** Adjustments to ongoing clinical trials (*N* = 14) and measures taken against COVID-19.

Implemented measure	Trials affected by each measure	
	*N*	%
Communication with trial sites		
Online meetings	14	100
Classrooms	14	100
Newsletters	14	100
Reports of the exceptional actions undertaken		
Conversion of physical visits into telehealth visits	6	42.9
Visit postponement	5	35.7
Visit cancellation	2	14.3
Patient transfer to other sites for		
Imaging	1	7.1
Surgery	1	7.1
Changes to ongoing trials		
Temporary halt		
In all recruitment sites	1	7.1
In part of the recruitment sites	4	28.6
Postponed activation of new recruitment sites	2	14.3
Study treatment interruption	0	0
Home drug delivery	0	0
Keeping trial integrity		
Implementation of remote monitoring	7	50.0
Case Report Forms update with COVID-19 infections	14	100
Follow-up of COVID-19 cases	14	100
Communication with health authorities		
Initiation of a new trial	1	7.1
Modification of pre-existing informed consent sheet	1	7.1

## Measures for clinical trial integrity

Communications with 250 principal investigators leading trials in eight European countries were strengthened through scheduled online meetings, chat rooms, and specific newsletters providing real-time information on the impact of the pandemic on clinical trial recruitment and follow-up (Table 1). Some investigators were reluctant to predict timelines for a return to normal operations because of the probable prolonged effects of the COVID-19 pandemic in many regions. At this time, a total of 329 patients (of whom 101 recruited during the pandemic) are receiving study treatment as planned, without significant difficulties in drug supply, and a similar number are being overseen for safety and efficacy after completing trial treatment with adjustments in follow-up and physical visits schedules (i.e., visit postponement, cancellation, or conversion to phone/video calls) according to clinical trial requirements and principal investigators advice (Table 1). Collecting biological samples continues in all active clinical trials, though specific measures, such as favoring local storage, limiting shipments, and modifying packaging to minimize the risk of infection have been implemented in ongoing clinical trials with enrolled patients, restrictions on shipment of biological samples were checked in all countries, the UK was the only one reporting limitations.

In line with the recently reported guidance for protocol modifications by the US Food and Drug Administration^8^, changes in monitoring plan to allow remote visits or postpone them and larger amounts of drug supply were implemented in seven actively recruiting studies. In the remaining cases, the original monitoring plan already included the possibility to perform remote visits or drug supply was not applicable. Informed consent form remained unchanged, with the exception of one study where patients could confirm their participation in the study remotely.

According to the European Medicines Agency’s guidance^3^, MEDSIR has performed longitudinal studies to assure that data previously collected remains meaningful. In these studies, follow-up visits for each clinical trial patient are reviewed, and patient and caregiver safety are always ensured.

Finally, new clinical trials continue to be started. One example is the COPERNICO study (NCT04335305) which aims to evaluate the efficacy of immune blocking in hospitalized patients with severe COVID-19 respiratory syndrome. And, the MEDSIR pipeline is moving forward toward having novel patient-centric therapeutic options at our fingertips, to ensuring continuing clinical cancer research and stimulating its further innovation.

Overall, clinical research and patient care are being disrupted in much of the world because of the most challenging health crises in modern history, causing difficulty with enrollment and completion of clinical trials. Our experience tells us that carrying out clinical research with certain adaptations to the COVID-19 pandemic is feasible, though difficult. Notwithstanding, we firmly believe that clinical research over standard care is necessary above all during this global public health crisis, putting the patients with poor prognoses first while offering them innovative and life-saving treatment options.
